# Joint Prevalence of Diabetes, Impaired Glucose Regulation, Cardiovascular Disease Risk and Chronic Kidney Disease in South Asians and White Europeans

**DOI:** 10.1371/journal.pone.0055580

**Published:** 2013-01-30

**Authors:** Kamlesh Khunti, Danielle H. Morris, Claire L. Weston, Laura J. Gray, David R. Webb, Melanie J. Davies

**Affiliations:** 1 Department of Health Sciences, University of Leicester, Leicester Diabetes Centre, Leicester General Hospital, Leicester, Leicestershire, United Kingdom; 2 Department of Cardiovascular Sciences, University of Leicester, Leicester Diabetes Centre, Leicester General Hospital, Leicester, Leicestershire, United Kingdom; German Diabetes Center, Leibniz Center for Diabetes Research at Heinrich Heine University Duesseldorf, Germany

## Abstract

**Background:**

Multiple vascular risk factors may confer very high risk, but the degree of commonality between risk factors is unclear, particularly among ethnic minorities. Furthermore, it is unknown what impact this commonality will have on the UK-based NHS Health Check Programme; a vascular disease prevention programme that screens individuals aged 40–74 years. We estimated the joint prevalence of diabetes, impaired glucose regulation (IGR), high cardiovascular disease (CVD) risk and chronic kidney disease (CKD) among White Europeans and South Asians who would be eligible for the Programme.

**Methods:**

Cross-sectional data were analysed for 3707 participants (23.6% South Asian) in a screening study set in Leicestershire, UK. Diabetes and IGR were screen-detected. CKD may have been diagnosed previously. IGR was defined as impaired fasting glucose and/or impaired glucose tolerance, and high CVD risk as 10 year risk greater than 20%.

**Results:**

Among males, South Asians had higher prevalence than White Europeans of diabetes (9.0% vs. 3.9%, respectively, p<0.001), IGR (12.5% vs. 9.2%, p = 0.06), and high CVD risk (39.1% vs. 33.1%, p = 0.03), but lower prevalence of CKD (1.5% vs. 4.6%, p<0.01). Among females, South Asians had higher prevalence than White Europeans of diabetes (7.4% vs. 3.3%, p<0.001), but lower prevalence of CKD (3.7% vs. 13.0%, p <0.001) and CVD risk (2.4% vs. 4.6%, p = 0.03), and a non-significant difference in IGR prevalence. At least one risk factor was diagnosed in 34% of participants, and all of them in 0.4%, suggesting that 723,589–734,589 more individuals each year will require monitoring following implementation of the Health Check Programme.

**Conclusions:**

The collective prevalence of risk factors for vascular disease in this population was high, but there was little overlap between the risk factors, and prevalence differed by ethnicity. This has implications for service delivery and resources, and should be considered when planning screening and intervention programmes.

## Introduction

Whilst the cumulative atherogenic effect of vascular risk factor clustering is well recognised, the degree of commonality of individual components within large populations is less clear, particularly among mixed ethnic populations, despite the well-established associations between ethnicity and vascular disease. This is important because it may identify different groups of people at increased risk, facilitate identification of groups at very high risk, and drive efficiency by enabling targeted screening. With earlier identification of vascular risk factors it may be possible to control the rate at which vascular disease progresses by modification of co-existing risk factors such as smoking, obesity, hypertension, and hyperglycaemia [1].

In the UK, the degree of commonality of vascular risk factors is also of interest due to the introduction of the NHS Health Check Programme. The Programme screens people aged 40 to 74 years without a history of vascular disease with the aim of primary prevention of stroke, heart disease, diabetes and kidney disease [2]. It is estimated that the Programme could prevent 1600 heart attacks and strokes, 4000 cases of diabetes, and 650 deaths each year [2]. Moreover, at least 20,000 cases of diabetes or kidney disease could be diagnosed earlier allowing individuals to be better managed and to improve their quality of life [2].

The aim of this study was to estimate the individual and joint prevalence of screen-detected type 2 diabetes, impaired glucose regulation (IGR; defined by impaired fasting glucose and/or impaired glucose tolerance), high cardiovascular disease risk (CVD; defined as 10 year risk greater than 20%), and chronic kidney disease (CKD) as vascular risk factors in White Europeans and South Asians screened as part of a population based diabetes screening programme (the ADDITION-Leicester study). As a secondary aim, we used these prevalence estimates to give an indication of the expected burden of new risk factors identified by the NHS Health Check Programme. It is vital to have accurate estimates of the expected burden of new disease so that commissioners can plan for prevention and management of these chronic diseases.

## Methods

### Ethics

Ethical approval was obtained from the University Hospitals of Leicester (UHL09320) and Leicestershire Primary Care Research Alliance (64/2004) local research ethics committees, and all participants gave written informed consent.

### Participants

The ADDITION-Leicester study is a UK-based two phase programme of research (NCT00318032) offering population level screening followed by multifactorial intervention for people with screen-detected type 2 diabetes mellitus. The recruitment process is detailed elsewhere [3]–[6], and a brief description follows. All general practices in the Leicestershire and Rutland Strategic Health Authority were invited to participate in ADDITION-Leicester. Of 46 practices invited, 28 accepted(61%) but 8 of these were not eligible because the data extraction programme that we used to extract information about eligible patients was only compatible with EMIS and these practices used a software programme other than EMIS. Therefore, the study included 20 out of 46 practices (43%), and these practices cover urban, suburban and rural Leicestershire. Participating practices were asked to identify patients suitable for inclusion from their computer systems. This involved the practice staff conducting a search systematically with the inclusion and exclusion criteria for the study. The inclusion criteria were that participants must be aged 40–75 years inclusive if they were of White European ethnicity and 25–75 years inclusive if they were of Asian, Black or Chinese ethnicity. Exclusion criteria included previous diagnosis of diabetes, being housebound, presence of a terminal illness, active psychotic illness, pregnancy or lactation. A random sample of eligible individuals were sent an invitation pack, which included a letter explaining that the pack could be requested in Hindi, Gujarati, Urdu or Punjabi, and a pre-screening questionnaire. Those responding to this letter were invited to a screening appointment [4]. To increase screening uptake, participants were able to attend a screening session at the diabetes research centres based at the local hospitals, other general practices or on a mobile screening bus, as well as at their own general practice.

Of the participants screened in the ADDITION-Leicester study, we included in these analyses only those who would be eligible for the NHS Health Check Programme, i.e. those aged 40–74 inclusive without diagnosed existing vascular disease (myocardial infarction, stroke, angina, angioplasty, high blood pressure, high cholesterol, atrial fibrillation, heart failure, or peripheral vascular disease). We also only included people of White European or South Asian ethnicity in line with the analysis aims. Participants were asked to classify their ethnicity into one of the categories used in the national census with the White European group defined as people who identified themselves as being ‘White British’, ‘White Irish’ or ‘Any other White background’ and the South Asian group defined as people who identified themselves as being ‘Indian’, ‘Pakistani’, ‘Bangladeshi’, or ‘Any other Asian background’. People who identified themselves as being in one of four mixed ethnicity categories, Chinese, Chinese Other, Caribbean, African or any other Black background were not included in these analyses. People who have previously been diagnosed with CKD are not eligible for the NHS Health Check Programme but this information was not available in the ADDITION-Leicester study, and so these individuals could not be excluded from these analyses.

### Variables

During the screening appointment, participants had a 75 g oral glucose tolerance test, which included fasting and postprandial blood samples, and were asked to provide information relating to smoking status, alcohol consumption, previous medical history and family history of disease. Other biomedical data were also available including HbA1c. Further details are given elsewhere [4]. Socio-economic status was measured using Index of Multiple Deprivation (IMD) scores, which is a postcode-based measure of socio-economic status where a higher score indicates higher deprivation.

Participants were diagnosed with type 2 diabetes or IGR according to the World Health Organization criteria (2011). Diabetes was defined as fasting blood glucose≥7.0 mmol/l, 2-hour post glucose challenge≥11.1 mmol/l or HbA1c≥6.5%. Individuals with glucose results within the diabetes range on their first oral glucose tolerance test were invited for a repeat oral glucose tolerance test to confirm the diagnosis on a separate day. IGR was defined as impaired fasting glycaemia (fasting blood glucose between 6.1 and 6.9 mmol/l) or impaired glucose tolerance (2-hour post glucose challenge between 7.8 and 11.0 mmol/l) [7].

Participants with an (MDRD calculated) eGFR value of less than 60 were defined as having CKD stage 3 or more. This degree of renal dysfunction has been shown to carry a significant risk of CVD risk mortality [8].

CVD risk was calculated using the Ethrisk score [9]. This formula assesses risk according to sex, age, smoking status, systolic blood pressure, HDL and total cholesterol levels, and ethnicity. High CVD risk was defined as 10 year CVD risk greater than 20%.

To assess the overall burden of these risk factors and their commonality, participants were classed as having any of the risk factors if they had at least one of screen-detected diabetes, IGR, high CVD risk, or CKD, and all of the risk factors if they had diabetes/IGR, high CVD risk and CKD.

### Statistical Analysis

Prevalence of screen-detected type 2 diabetes, IGR, high CVD risk, CKD, ‘any risk factor’, and ‘all risk factors’ was calculated in the overall study population, as well as in ethnicity by sex subgroups. Chi-squared tests were used to compare risk factor prevalence by ethnic group separately for males and females, and logistic regression was used to compare prevalence between the two ethnic groups adjusted for age, sex and ethnic-specific body mass index groups. Body mass index categories were based on the ethnic specific cut-points defined by the World Health Organization (overweight: 25–30 g/m^2^ for White Europeans, 23–27.5 kg/m^2^ for South Asians; obese: >30 kg/m^2^ for White Europeans, >27.5 kg/m^2^ for South Asians) [10]. In the logistic regression, missing data for explanatory and outcome variables were replaced using multiple imputation (the number of missing values for each variable is shown in Tables 1 and 2).

**Table 1:** Demographics of the study population by ethnicity and sex. Data shown are count (percentage) unless specified.

	Males (n = 1728)			Females (n = 1979)			
Characteristic	White European	South Asian	P^a^	White European	South Asian	P^a^	Total
Age^b^, years							
40–44	190 (14.3)	90 (22.4)		220 (14.6)	118 (25.0)		618 (16.7)
45–49	207 (15.6)	103 (25.6)		215 (14.3)	113 (23.9)		638 (17.2)
50–54	151 (11.4)	75 (18.7)		190 (12.6)	96 (20.3)		512 (13.8)
55–59	270 (20.4)	55 (13.7)		315 (20.9)	83 (17.6)		723 (19.5)
60–64	228 (17.2)	40 (10.0)		261 (17.3)	40 (8.5)		569 (15.4)
65–69	169 (12.8)	22 (5.5)		173 (11.5)	15 (3.2)		379 (10.2)
70–74	111 (8.4)	17 (4.2)	<0.001	133 (8.8)	7 (1.5)	<0.001	268 (7.2)
Mean (SD)	56.1 (9.2)	51.7 (8.4)	<0.001	56.1 (9.2)	50.8 (7.4)	<0.001	54.9 (9.2)
Smoking status^b^							
Non-Smoker	583 (44.3)	283 (71.1)		849 (56.8)	470 (99.6)		2185 (59.3)
Current Smoker	265 (20.1)	70 (17.6)		254 (17.0)	2 (0.4)		591 (16.1)
Ex-Smoker	469 (35.6)	45 (11.3)	<0.001	393 (26.3)	0 (0.0)	<0.001	907 (24.6)
Body mass index^c^, kg/m^2^							
Normal	310 (23.4)	88 (21.9)		535 (37.2)	79 (16.7)		1012 (27.3)
Overweight	682 (51.4)	179 (44.5)		561 (37.2)	172 (36.4)		1594 (43.0)
Obese	323 (24.4)	134 (33.3)		404 (26.8)	220 (46.6)		1081 (29.2)
Missing	11 (0.8)	0 (0.0)	0.003	7 (0.5)	1 (0.2)	<0.001	20 (0.5)
Mean (SD)	27.8 (4.1)	26.3 (4.2)	<0.001	27.5 (5.3)	27.6 (4.9)	0.722	27.5 (4.8)
Mean (SD) IMD score	16.5 (12.0)	22.8 (12.4)	<0.001	16.9 (11.8)	23.9 (12.9)	<0.001	18.2 (12.4)
Total	1326 (100.0)	402 (100.0)		1507 (100.0)	472 (100.0)		3707 (100.0)

Abbreviations: IMD, Index of Multiple Deprivation; SD, Standard Deviation.

a P-values show the difference between White European and South Asians within each sex group, and were calculated using X^2^ tests for categorical variables and t-tests for continuous variables.

b There were no missing data for these variables.

c Body mass index categories were based on ethnic specific cut-points, as follows: 25–30 kg/m^2^ for White Europeans and 23–27.5 kg/m^2^ for South Asians were defined as overweight, and >30 kg/m^2^ for White Europeans and >27.5 kg/m^2^ for South Asians were defined as obese [10].

**Table 2:** Prevalence of screen-detected type 2 diabetes, impaired glucose regulation (IGR), high cardiovascular disease (CVD) risk and chronic kidney disease (CKD) by sex and ethnicity.

	Males			Females			
	White European	South Asian	P^a^	White European	South Asian	P^a^	All participants
Diabetes	52/1323	36/401	<0.001	49/1503	35/471	<0.001	172/3698
	3.9 (2.9, 5.0)	9.0 (6.2, 11.8)		3.3 (2.4, 4.2)	7.4 (5.1, 9.8)		4.7 (4.0, 5.3)
IGR	122/1321	50/400	0.057	140/1501	51/471	0.337	363/3693
	9.2 (7.7, 10.8)	12.5 (9.3, 15.7)		9.3 (7.9, 10.8)	10.8 (8.0, 13.6)		9.8 (8.9, 10.8)
High CVD risk^b^	426/1289	152/389	0.028	67/1451	11/462	0.034	656/3591
	33.1 (30.5, 35.6)	39.1 (34.2, 43.9)		4.6 (3.5, 5.7)	2.4 (1.0, 3.8)		18.3 (17.0, 19.5)
CKD	61/1314	6/399	0.005	194/1493	17/464	<0.001	278/3670
	4.6 (3.5, 5.8)	1.5 (0.3, 2.7)		13.0 (11.3, 14.7)	3.7 (2.0, 5.4)		7.6 (6.7, 8.4)
Any risk factor^c^	544/1298	199/394	0.003	397/1467	106/464	0.071	1246/3623
	41.9 (39.2, 44.6)	50.5 (45.6, 55.4)		27.1 (24.8, 29.3)	22.8 (19.0, 26.7)		34.4 (32.8, 35.9)
All risk factors^c^	10/1320	1/400	0.265	2/1499	0/469	0.429	13/3688
	0.8 (0.3, 1.2)	0.3 (0.0, 0.7)		0.1 (0.0 to 0.3)	0		0.4 (0.2, 0.5)

Data shown are number of cases/total and percentage (95% confidence interval).

Missing values: Diabetes = 9; IGR = 7; CVD risk = 116; CKD = 37; Any = 48; All = 11.

a P-values were estimated using X^2^ tests and show the difference in prevalence between White Europeans and South Asians for each sex.

b High CVD risk was defined as a risk score greater than 20%.

c Any risk factor means that the person has at least one of diabetes, IGR, high CVD risk or CKD. All rick factors means that the person has diabetes or IGR, high CVD risk and CKD.

Finally, we used our prevalence estimates to approximate the number of new cases of type 2 diabetes, high CVD risk and CKD that would be diagnosed each year by the NHS Health Check Programme, assuming that the screening uptake was 75% which means that 2.2 million people will be screened each year [11]. We standardised our estimates to the ethnic, age and sex distribution of England [12]. We did this by estimating the expected number of people who would be screened in each ethnic, 5-year age, and sex group, and then used the risk factor prevalence within the respective group to estimate the number of expected diagnoses within that group. For example, 8.01% of the eligible population (ages 40–74) were male, of White ethnicity and aged 40–44 years, so if 2.2 million people were screened then we would expect 176,220 of those screened to be in this group. Screen-detected diabetes prevalence was 1.58% among White men aged 40–44 and so we would expect 2784 (1.58% of 176,220) diabetes cases to be diagnosed in this group. The number of expected diagnoses in each of the groups was summed to provide the overall estimate. We did not have prevalence estimates for other ethnic groups, and so we assumed that the prevalence in those groups was the same as that in the White European or the South Asian population in turn, which produced a range of plausible estimates. We repeated these analyses assuming that there is a 45% uptake to screening similar to the rate in Dalton *et al* [13].

Data were analysed using SAS version 9.2 (SAS Institute Inc. Cary, NC, USA). Statistical significance was assessed at the 5% level and precision of the estimates is represented by 95% confidence intervals. All p-values shown are two-sided.

## Results

### Description of the ADDITION-Leicester study population

**Figure 1:**
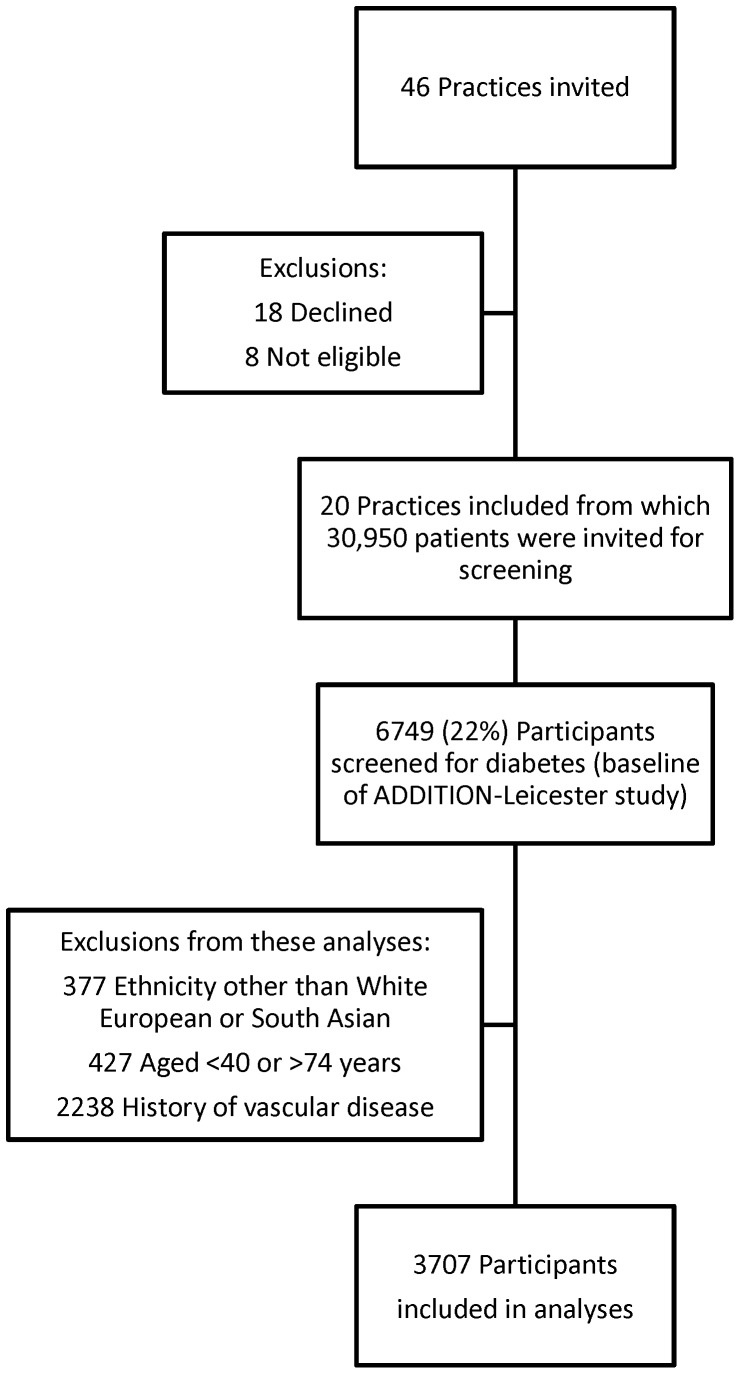
Flow of Participants. The Figure shows the flow of participants into the ADDITION-Leicester study and the current analyses.

Figure 1 shows the flow of ADDITION-Leicester participants into these analyses. A total of 6749 participants were screened in ADDITION-Leicester, 3042 of whom were not eligible for these analyses meaning that 3707 participants were included (874 South Asians and 2833 White Europeans).

The demographic characteristics of the study population are shown in Table 1. South Asians were younger compared with White Europeans on average (mean age 51.7 vs. 56.1 years, respectively, p<0.001 for males; mean age 50.8 vs. 56.1 years, respectively, p<0.001 for females). Smoking status also differed significantly by ethnicity (p<0.001 for both sexes). Although the proportion of current smokers was similar for South Asian and White European men, White European men were more likely to be ex-smokers (35.6%) whereas South Asian men were more likely to be non-smokers (71.1%). Only two South Asian women were classed as current smokers. Ethnic-specific body mass index cut-points resulted in a higher proportion of South Asians classified as obese (33.3% vs. 24.4%, p<0.01, and 46.6% vs. 26.8%, p<0.001, for males and females, respectively), even though mean BMI was lower for South Asian men compared with White European men (26.3 kg/m^2^ vs. 27.8 kg/m^2^, p<0.001) and was similar among White European and South Asian women (p = 0.72). Deprivation scores were significantly higher in South Asians compared with White Europeans (p<0.001 for both males and females).

### Prevalence of the risk factors within ADDITION-Leicester

Overall, 172 (4.7%) individuals had screen-detected diabetes, 363 (9.8%) had IGR, 656 (18.3%) were at high CVD risk, and 278 (7.6%) had CKD (Table 2). Among males, South Asians had a higher prevalence of screen-detected diabetes (p<0.001), IGR (p = 0.06), and high CVD risk (p = 0.03), but a lower prevalence of CKD (p<0.01) compared with White Europeans. Among females, South Asians had a higher prevalence of screen-detected diabetes (p<0.001), but White Europeans had a higher prevalence of CKD (p<0.001) and CVD risk (p = 0.03), and there was no difference in prevalence of IGR between the ethnic groups (p = 0.34). After adjustment for age, body mass index and sex (Table 3), South Asians were still more likely than White Europeans to be diagnosed with diabetes (p<0.001), IGR (p = 0.01), and high CVD risk (p<0.001), but less likely to be diagnosed with CKD (p<0.001).

**Table 3:** Unadjusted and adjusted differences in risk factor prevalence between White Europeans and South Asians.

	Odds of risk factor being diagnosed in South Asians compared with White Europeans			
	Unadjusted		Adjusted^a^	
Condition	OR (95% CI)	P value	OR (95% CI)	P value
Diabetes	2.39 (1.74, 3.27)	<0.001	2.54 (1.81, 3.56)	<0.001
IGR	1.28 (1.00, 1.64)	0.047	1.40 (1.08, 1.81)	0.012
High CVD risk^b^	1.09 (0.89, 1.33)	0.396	4.05 (2.95, 5.56)	<0.001
CKD	0.28 (0.18, 0.43)	<0.001	0.36 (0.23, 0.56)	<0.001
Any risk factor^c^	1.06 (0.91, 1.25)	0.445	1.87 (1.54, 2.26)	<0.001

Abbreviations: CI, Confidence Interval; CKD, Chronic Kidney Disease; CVD, Cardiovascular disease; IGR, Impaired Glucose Regulation; OR, Odds Ratio.

Note: For all explanatory and outcome variables, missing values were replaced using multiple imputation methods so the results in this Table are based on all 3707 participants. Too few people were diagnosed with all outcomes to allow for reasonable estimates to be modelled.

a Odds ratios were adjusted for age, body mass index, and sex.

b High CVD risk was defined as a risk score greater than 20%.

c Any risk factor means that the person has at least one of diabetes, IGR, high CVD risk or CKD.

### Any or all of the risk factors within ADDITION-Leicester

A total of 1246 individuals (34.4%) were diagnosed with at least one of screen-detected diabetes, IGR, high CVD risk, or CKD (Table 2). South Asian men were more likely to have one of the risk factors (p<0.01) whereas South Asian women were less likely to have a risk factor (p = 0.07) compared with their White European counterparts. South Asians remained at a higher risk of having at least one of the risk factors after adjustment for sex, age, and body mass index (p<0.001, Table 3).

**Figure 2:**
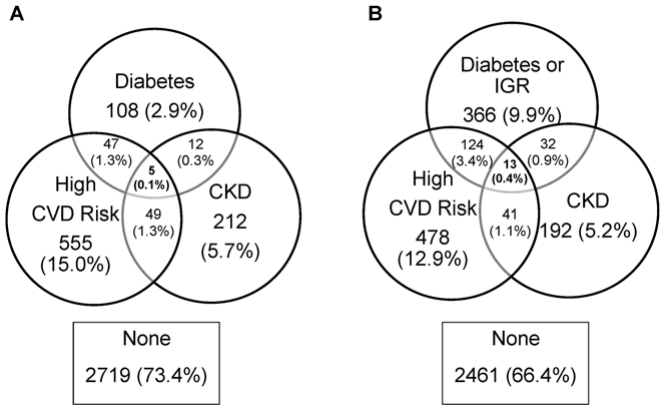
Joint Prevalence of Vascular Risk Factors. The Figure shows the joint prevalence of screen-detected diabetes, impaired glucose regulation (IGR), high cardiovascular disease (CVD) risk, and chronic kidney disease (CKD) in a UK-based screening study. Note that these diagrams assume that those with missing data do not have the risk factor(s) for which data are unavailable. Panel A shows the overlap between diabetes, high CVD risk and CKD. Panel B shows the overlap between diabetes or IGR, high CVD risk and CVD.

**Figure 3:**
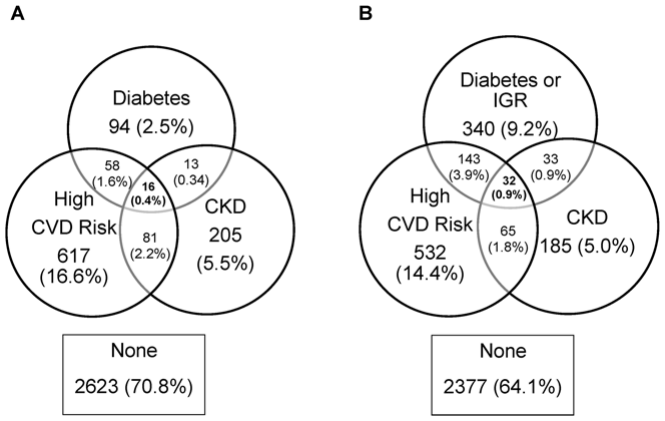
Joint Prevalence of Vascular Risk Factors with Sensitivity Analysis for Missing Data. Sensitivity analysis showing the joint prevalence of screen-detected diabetes, impaired glucose regulation (IGR), high cardiovascular disease (CVD) risk, and chronic kidney disease (CKD) assuming that those with missing data have the risk factor(s) for which data are unavailable. Panel A shows the overlap between diabetes, high CVD risk and CKD. Panel B shows the overlap between diabetes or IGR, high CVD risk and CVD.

Figure 2(a) shows the overlap between prevalence of screen-detected diabetes, high CVD risk and CKD, and Figure 2(b) shows the same information with IGR included in the definition of diabetes. Diagnosis of all risk factors was rare with only 13 participants (0.4%) found to have diabetes/IGR, high CVD risk and CKD. Based on this small number of events, differences in prevalence between ethnic groups tended not to be significant (Table 2). Figure 3 shows the same information as Figure 2 with a different assumption regarding missing data. It can be seen that the degree of overlap was similar regardless of which assumptions were used.

### Expected number of diagnoses identified by the NHS Health Check Programme based on extrapolated data

If 2.2million people are screened each year (∼75% uptake) then it is estimated that the number of people who will be diagnosed will be between 84,038 and 89,231 for type 2 diabetes, between 381,855 and 390,602 for CVD risk, between 181,320 and 184,752 people for CKD, and between 578,954 and 587,631 for any of these three risk factors. An estimated 203,135 to 206,753 people will be diagnosed with IGR each year, and between 723,589 and 734,589 people with any of diabetes/IGR, CVD risk, and CKD. If 1,350,000 people are screened each year (45% uptake) then the estimated number of new diagnoses each year is 51,569–54,755 for type 2 diabetes, 234,320–239,687 for CVD risk, 111,265–113,370 for CKD, 124,651–126,871 for IGR, and 444,020–450,471 for any of diabetes/IGR, CVD risk, and CKD.

## Discussion

### Principal findings

In our population of over 3500 White European and South Asian individuals who were previously undiagnosed and would be eligible for the NHS Health Check Programme, the overall prevalence of screen-detected diabetes was approximately 5%, of IGR was approximately 10%, of high CVD risk was 18%, and of CKD was 8%. Furthermore, 34% of individuals had at least one of these risk factors for vascular disease, and only 0.4% had all of these risk factors. Generally, risk factor prevalence was higher among South Asians than White Europeans, except for CKD.

### Strengths and limitations of the study

A strength of our study was that it was conducted on a cohort with a high proportion of individuals of South Asian origin. Whilst we acknowledge that the utility of ethnicity as a concept is limited, it is well-established that there are ethnic differences in the risk of certain diseases such as diabetes due to genetic and cultural risk factors. Furthermore, ethnicity-related inequalities in health care continue to exist. Understanding the prevalence of vascular risk factors in non-White groups is therefore highly important as these differences might reflect underlying biological mechanisms, but might also reflect differences in contact with the health care system.

A major limitation of our study is that it suffered from a low response rate with just over 20% of eligible patients attending screening appointments [6], and so is likely subject to some degree of selection bias. There may be a number of reasons for this but it is likely that the time consuming process of attending an oral glucose tolerance test deterred many people from attending the screening appointment. Also, many eligible patients were from deprived areas and/or ethnic minority populations which may have affected participation rates. The extent of the selection bias is probably small because the response rate is similar to other studies conducted in deprived areas or in people of minority ethnic groups [14], the patient characteristics of those participating in the study are similar to those of the Leicester population [6] (for example, 24% of the study population was South Asian compared with 27% of adults in Leicester [12]), and 18% of individuals were found to have a high CVD risk, a finding similar to that suggested in the NHS Health Check Programme [2], suggesting that similar selection biases are present in our study to those in the Programme. People who take part in studies tend be healthier than the general population. Thus, the likely consequence of a selection bias is that our prevalence estimates for the individual risk factors will be underestimated, which could also result in an underestimation of the overlap between the risk factors. It is unclear what the consequence of this would be on our estimates at the national level as it would depend on how much the overlap increased in relation to the individual components increasing.

We held detailed medical history for the study participants and so were able to closely replicate the inclusion criteria for the NHS Health Check Programme. However, we were unable to exclude people with previously diagnosed CKD and so our estimates of undiagnosed CKD might be artificially inflated. This is unlikely to affect the comparison between ethnic groups in terms of kidney disease prevalence.

A potential drawback of our study is that we used only one risk score (Ethrisk) to identify people at a high risk of CVD when there are several such measures available. NICE do not currently recommend one CVD risk score over another but the most commonly used ones are Framingham, Ethrisk (which is based on Framingham but with an ethnicity correction), and QRisk. It appears that Framingham overestimates CVD risk in comparison with QRisk, which would not affect the relative comparisons within our study, but could potentially have inflated the absolute estimates.

Multiple imputation, rather than complete case analysis, was used as both lead to negligible biases when data are missing at random, but multiple imputation is more efficient. Furthermore, the most data that are missing for any one variable is 3% so the multiple imputation is likely to have had a negligible effect on our findings.

Finally, the vast majority of the participants in ADDITION-Leicester were of White European or South Asian ethnicity; therefore, we could not reasonably estimate the prevalence of risk factors in other ethnic groups and so these individuals were excluded from the present analyses. A consequence of this is that when we standardised our prevalence rates to the UK population we had to make an assumption that people of other ethnicities had risk factor prevalence that was similar to White Europeans, South Asians or somewhere in between. If in fact the prevalence is much higher or much lower among other ethnic groups then our extrapolations may not be reasonable. Nevertheless, any effect is likely to be small because altogether these groups only comprise a small percentage (6.2%) of the UK population.

### Comparison with other studies

The ethnic composition of our study population allowed us to investigate the prevalence of various vascular risk factors in a population that comprised a higher proportion of South Asians than previous studies. Our prevalence estimates for the individual risk factors that we considered are in agreement with existing estimates [16]–[19]. There are less data available for the commonality of these risk factors. In our study, while many people had at least one risk factor, few people had all of them. Only approximately 10% of the people with screen-detected diabetes also had CKD. This estimate is much lower than that observed in other studies [20], [21]. This might be because there were a higher proportion of South Asians in our study than in previous studies, and South Asians had a lower prevalence of CKD than White Europeans, or because these diseases might develop during different time frames and so are not all present cross-sectionally, or because only screen-detected diabetes was included whereas the overlap might have been greater had prevalent diabetes also been considered.

There are several possible reasons why South Asians had a lower prevalence of CKD than White Europeans. For example, it might be because we used the MDRD equation to estimate GFR which might underestimate CKD among South Asians [22]. We chose to use the MDRD equation, rather than alternatives such as CKD-EPI, because MDRD is most commonly used in clinical practice. However, it is a known limitation of all eGFR equations that currently none have been adequately validated in South Asian populations and tend to underestimate CKD. An alternative explanation for the differences in CKD prevalence is related to ethnic differences in diabetes prevalence. GFR tends to be raised in people with newly diagnosed Type 2 diabetes compared with people with normoglycaemia, and so it might be expected that people diagnosed with diabetes through the NHS Health Check Programme would be at a reduced risk of CKD.

We observed greater overlap between high CVD risk and both screen-detected diabetes and CKD. This is consistent with the increased risk of CVD observed in people with diabetes and/or CKD [14], and with the observed association between risk markers of CKD [3], [15] and cardiovascular events.

### Clinical relevance

Our findings have important implications for the NHS Health Check Programme, which was introduced by the Department of Health in April 2009 [2]. From our population sample of 6749 individuals, 33% were excluded because they had had a previous cardiovascular event or some other condition that means they should already be monitored through an existing care pathway. This number is in fact slightly higher because people with diabetes were already excluded from our population sample. It is possible that the people who attended screening were more likely to have a pre-existing condition. Nevertheless, this high percentage of exclusions indicates a high burden of existing monitoring that is likely to be substantially increased by the Programme. The low degree of risk factor commonality in our study suggests that a greater number of individuals will require management than if the degree of commonality was high. Our projections for the number of new diagnoses of diabetes, high CVD risk and CKD are much higher than the previously quoted figure of at least 20,000 diagnoses [2], even when the projections were based on a screening uptake of only 20%. This might in part be a consequence of us being unable to exclude people with previously diagnosed CKD from our analyses since this information was not collected in the ADDITION-Leicester study. However, the effect of this is likely to be small because our projections for diabetes and CVD risk only varied by a few thousand when we used the extreme assumption that all of the people that were diagnosed with CKD in ADDITION-Leicester were already known to have the disease and thus would be ineligible for the NHS Health Check Programme (data not shown). Our higher projections suggest that the burden of risk factors diagnosed though the NHS Health Check Programme on primary care trusts might be greater than previously anticipated.

The NHS Health Check Programme offers a real opportunity to make significant contributions to changing health inequalities, including ethnic, socio-economic and sex inequalities. However, it requires individual primary care trusts to ensure that their approach is appropriate for their own community [23]. In view of the differences in risk factor prevalence between ethnic groups that were highlighted in our study, this is particularly important in a population comprising different ethnic communities where individuals may not get the services they need because of differences in language, literacy and culture [24]. Screening of individuals at risk of vascular disease will thus need to be adapted to provide for individual needs. It may be that part of the screening programme ensures that individuals are aware of their ethnic-specific risks so that they are more likely to access screening programmes. Individual ethnic groups may also need tailored intervention programmes. For example, systematic reviews have shown that levels of physical activity are lower in all South Asian groups [25] and so it may be beneficial to advocate exercise within this community.

### Future research and Summary

Among White Europeans and South Asians, the prevalence of screen-detected diabetes, IGR, high CVD risk and CKD is high, but there is little overlap between these vascular risk factors. Future research should focus on outcomes in the group of individuals who have all of these risk factors as they are potentially a very high risk group. Additionally, we have highlighted ethnic differences in prevalence of vascular risk factors. Our findings suggest that the burden of risk factors diagnosed through the NHS Health Check Programme on general practitioners may be greater than previously expected, and emphasise the importance of offering the Programme in a wide range of settings with a view to decreasing health inequalities between ethnic groups.
